# In Vivo Biocompatibility of Electrospun Biodegradable Dual Carrier (Antibiotic + Growth Factor) in a Mouse Model—Implications for Rapid Wound Healing

**DOI:** 10.3390/pharmaceutics11040180

**Published:** 2019-04-14

**Authors:** Charu Dwivedi, Himanshu Pandey, Avinash C. Pandey, Sandip Patil, Pramod W. Ramteke, Peter Laux, Andreas Luch, Ajay Vikram Singh

**Affiliations:** 1 Department of Biological Sciences, Sam Higginbottom University of Agriculture, Technology and Sciences, Allahabad 211007, India; charucas0505@gmail.com; 2 Nanotechnology Application Centre, Faculty of Science, University of Allahabad, Allahabad 211002, India; himanshu.nac@gmail.com (H.P.); prof.avinashcpandey@gmail.com (A.C.P.); 3 Department of Pharmaceutical Sciences, Faculty of Health Sciences, Sam Higginbottom University of Agriculture, Technology & Sciences, Allahabad 211007, India; 4 E-Spin Nanotech Pvt Ltd., Kanpur 208016, India; espinnanotech@gmail.com; 5 Department of Chemical and Product Safety, German Federal Institute for Risk Assessment (BfR), Max-Dohrn-Strasse 8-10, 10589 Berlin, Germany; peter.laux@bfr.bund.de (P.L.); andreas.luch@bfr.bund.de (A.L.); 6 Physical Intelligence Department, Max Planck Institute for Intelligent Systems, 70569 Stuttgart, Germany

**Keywords:** tissue engineering, growth factor, diabetic, wound healing, nanocomposite

## Abstract

Tissue engineering technologies involving growth factors have produced one of the most advanced generations of diabetic wound healing solutions. Using this approach, a nanocomposite carrier was designed using Poly(d,l-lactide-*co*-glycolide) (PLGA)/Gelatin polymer solutions for the simultaneous release of recombinant human epidermal growth factor (rhEGF) and gentamicin sulfate at the wound site to hasten the process of diabetic wound healing and inactivation of bacterial growth. The physicochemical characterization of the fabricated scaffolds was carried out using scanning electron microscopy (SEM) and X-ay diffraction (XRD). The scaffolds were analyzed for thermal stability using thermogravimetric analysis and differential scanning calorimetry. The porosity, biodegradability, and swelling behavior of the scaffolds was also evaluated. Encapsulation efficiency, drug loading capacity, and in vitro drug release were also investigated. Further, the bacterial inhibition percentage and detailed in vivo biocompatibility for wound healing efficiency was performed on diabetic C57BL6 mice with dorsal wounds. The scaffolds exhibited excellent wound healing and continuous proliferation of cells for 12 days. These results support the applicability of such systems in rapid healing of diabetic wounds and ulcers.

## 1. Introduction

Wound healing or tissue repair is a complex multistep process comprising a multitude of cells, cytokines, extracellular matrix (ECM) molecules, blood cells, and a number of other factors [1]. Disturbances at proliferation or inflammation phases of wound healing lead to perturbations in the process [2]. Chronic wounds, such as those in diabetic patients, present worldwide health challenges as well as an economic burden. The existing therapies exhibit unsatisfactory results and despite treatment of these chronic wounds, which involves tight glucose control and meticulous wound care, the prognosis for their healing is quite poor and finally leads to morbidity [3,4]. Microorganisms are naturally present on the wound bed without any detrimental effects to the normal wound healing process since the number of bacteria is quite low. Since diabetes lowers the normal immunity of the body, such patients are more prone to infection in wounds [5]. Hence, chronic wounds, such as leg ulcers, foot ulcers, and pressure ulcers, are more prone to such microbial infections due to the presence of multiple microbial populations consisting of *S. aureus* [6], *P. aeruginosa* [7], coliform bacteria [8], *Streptococcus* spp., and *Enterococcus* spp. [9]. During injury, foreign bodies including pathogenic microbes can enter deep into the wounds causing chronic inflammatory responses and, thereby, delaying healing of the wounds. The patient may develop a more serious deep-wound infection, and require amputation due to the occurrence of anaerobic bacteria in diabetic foot ulcers and may also lead to abscess or granuloma formation [10,11].

Generally, the process of wound healing is driven by many cellular mediators including various growth factors which are proteinaceous molecules playing a key role in tissue repair and wound care [12]. One of the major obstacles in the treatment method involving growth factors is that the encapsulated growth factor tends to be released in a non-controllable manner due to its physical association with the drug reservoir systems [13]. Moreover, the growth factors are either easily degraded by proteinases or removed by exudate before reaching the wound bed. Until now, only platelet-derived growth factor-BB (PDGF-BB) has successfully passed clinical trials [1]. Epidermal growth factor (EGF) is a low-molecular-weight polypeptide and plays a significant role in wound healing as it stimulates proliferation, differentiation, and survival of cells [14]. Hence, recombinant human epidermal growth factor (rhEGF) was selected as the bioactive agent for immobilization on the nanofibrous scaffolds. It acts by binding with high affinity to epidermal growth factor receptor (EGFR) on the cell surface and stimulating the intrinsic protein-tyrosine kinase activity of the receptor. The tyrosine kinase activity, in turn, initiates a signal transduction cascade that results in a variety of biochemical changes within the cell—a rise in intracellular calcium levels, increased glycolysis and protein synthesis, and increases in the expression of certain genes including the gene for EGFR—that ultimately lead to DNA synthesis and cell proliferation [15].

In the recent scenario, due to their comprehensive assortment of implementations in the world of biomedicine, nanomaterials have emerged as potent tools for clinicians and researchers in different biomedical and allied fields of human life [16,17,18]. Nanomaterials possess notable virtues, such as high reactivity, large surface-to-mass ratio, and ultra-small size making them highly useful in biomedical applications [19,20].

In view of these strategies, current tissue engineering approaches are centered around the fabrication of three dimensional (3D) nanoscaffolds or ECM analogs that should conform to multifactorial requirements, for example, those associated with tissue repair [16]. Such scaffolds tune the biomimetic nature of the ECM, possess large surface area to volume ratio, are able to facilitate diffusion (as a result of high porosity), and have tunability of physical properties simultaneously providing a local release of different biomolecules to address successful tissue regeneration [21]. Numerous studies have been done to fabricate potentially applicable scaffold materials for tissue engineering and wound healing applications. Electrospun nanofibrous scaffolds have been successfully used in site-specific delivery of many bioactive molecules and for the treatment of various infections and cancers. Such scaffolds allow for the release of loaded biomolecules in therapeutic dosage and have a negligible influence on drug activity and possess well-controlled drug release rate [22,23]. One of the smart property of these scaffolds is that they possess physical resemblance with ECM and are easy to implement due to their superior mechanical durability, flexibility in surface functionalities, and interconnected and readily controlled secondary structures [24]. Polymers had been a choice material for the fabrication of nanofibrous scaffolds. Synthetic biodegradable polymers, such as poly(3-caprolactone), polyethylene oxide, poly(l-lactide-*co*-3-caprolactone) (PLCL), polylactic acid, poly(lactide-*co*-glycolide), and polyglycolic acid (PGA), Poly(vinyl alcohol (PVA), Polyvinyl chloride (PVC), Poly-L-lactic acid (PLLA), nylon, gelatin, polyurethane, etc., have proven useful in the preparation of nanofibrous scaffolds. Researches have proved these scaffolds to be biocompatible and to enhance cell functions in vitro [25,26].

Cell functions include the growth, differentiation, and metabolism of cells via interaction with specific cell-surface receptors [27]. Polymers like Poly (d,l-lactide-*co*-glycolide) (PLGA) have been extensively used for application in drug-delivery, surgical implants, and tissue engineering scaffolds owing to their biodegradability and biocompatibility [28,29]. Further, it is imperative that the wound is free from infection-causing microbes and all factors which inhibit its natural healing process [30]. The addition of antibacterial agents into electrospun fibers has been a thrust area of research for clinicians especially as antibiotic-resistant bacteria strains increasingly emerge [31]. Gentamicin sulfate is a bactericidal aminoglycoside antibiotic that is widely used for the treatment of bacterial infections [32]. Electrospun nanofibers could increase the drug efficiency and drug solubility in aqueous solution due to their high surface-to-volume ratio. They could provide a controlled drug delivery to the site of action in the body in an optimal concentration-versus-time profile. A multitude of nanomaterials conjugated with vancomycin has been designed to enhance the pharmacokinetics and pharmacodynamics of the antibiotic molecule [31,33,34,35,36].

In this work, we fabricated a novel biomimetic drug and rhEGF delivery system by the electrospinning of PLGA/Gelatin solutions containing Gentamicin Sulfate (GS) and further immobilizing the scaffolds with rhEGF. The purpose of this work is to support diabetic wound healing and to eliminate microbial infections while releasing gentamicin and rhEGF in a controlled fashion. To our knowledge, synchronous release of drug and growth factor from electrospun scaffolds has not been investigated and we did not find any paper which discusses it. In the present scenario, controlled release of antibiotic and growth factor was achieved, and the dual delivery system allows for efficient and for the treatment of wounds in diabetic patients.

## 2. Experimental

### 2.1. Materials

Poly (d,l-lactide-*co*-glycolide) (LA/GA 85/15) (Gangwal Chemicals Pvt. Ltd., Mumbai, India), gentamicin sulfate (FDC India Ltd., Mumbai, India), streptozotocin (Sisco Research Laboratories Pvt. Ltd., Bhiwandi, India), nutrient broth and nutrient agar (Merck Specialities Pvt. Ltd. Mumbai, India), 2,2,2-trifluoroethanol, suberic acid bis(*N*-hydroxy-succinimide ester) (NHS), 1,6-diaminohexane and 2,2-dihydroxyindane-1,3-dione (ninhydrin) (Loba Chemie Pvt. Ltd. Mumbai, India) and rhEGF (Life Technologies India Pvt. Ltd., New Delhi, India). All other chemicals and reagents were of analytical grade as purchased.

### 2.2. Fabrication of GS-Loaded Composite PLGA/Gelatin Nanoscaffolds

**Figure 1:**
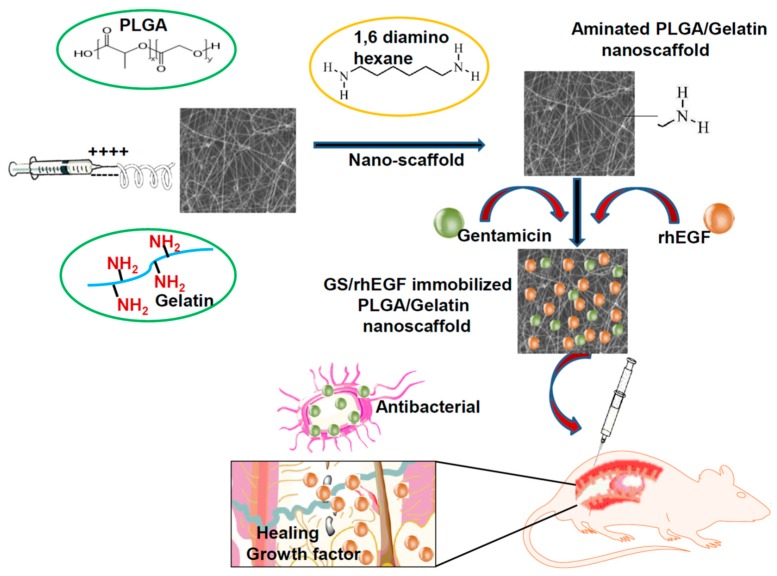
Process of fabrication of aminolyzed Poly(d,l-lactide-*co*-glycolide) (PLGA)/Gelatin nanoscaffolds and subsequent application on diabetic wounds.

Composite PLGA/Gelatin nanoscaffolds were fabricated as described in our previous study [21,37]. Schematic Figure 1 demonstrates the procedure for the fabrication of electrospun nanofibrous scaffolds, their immobilization with the growth factors (rhEGF and Gentamicin sulfate (GS) and their application on wounds of diabetic mice for in vivo test. Poly (d,l-lactide-*co*-glycolide) was used with LA/GA in the ratio 85/15 due to the fact that it takes an average degradation time of 5 to 6 weeks [38]. Briefly, two different composite polymer solutions of 10% PLGA/Gelatin (wt. ratio 70:30 and 50:50, respectively) in 2,2,2-trifluoroethanol were prepared. Thereafter, 5 mg/mL gentamicin sulfate and 0.2 mL of PEG-400 was added to each of the prepared polymer solutions, and were electrospun into nanofibrous scaffolds using an electrospinning setup. The electrospinning was operated at the flow rate of 0.6 mL/h, and a high voltage (16 kV) was applied to the tip of the needle (0.9 mm inner hole diameter) attached to the syringe. The ultrafine aligned nanofibers were collected by a rotating the disc wrapped with a piece of aluminum foil with a horizontal distance of 10 cm from the needle tip. The obtained electrospun nanofibrous scaffolds were dried at room temperature under vacuum for 24 h to remove organic solvent and moisture. The scaffolds were stored at −20 °C for further analysis.

### 2.3. Post-treatment of Composite Nanoscaffolds via Covalent Immobilization of rhEGF

The growth factor rhEGF was immobilized on the surface of the prepared nanoscaffolds by the covalent immobilization technique [21]. Before immobilization, the scaffolds were aminolyzed to introduce amine groups into the surface of the prepared scaffolds. Henceforth, the scaffold pieces (20 mm in diameter) were soaked into a 10% (*w*/*v*) solution of 1,6-hexanediamine prepared in isopropanol and were incubated at room temperature for 3 h and subsequently washed in PBS (phosphate-buffered saline, pH 7.4) five times. Then the scaffolds were rinsed with 70% ethanol and PBS. Thereafter, they were immersed in 10 mL PBS containing 0.08 mmol NHS with gentle agitation for 4 h at room temperature. The scaffolds were again rinsed gently five times with PBS to remove residual solvents and make sure that scaffolds are not removed from the substrate (Supplementary Material: Figure S1) five times with PBS. Finally, the activated scaffolds were soaked into 10 μg/mL rhEGF solutions in PBS with gentle shaking for 12 h at 4 °C. Then the rhEGF-immobilized composite nanoscaffolds were washed with PBS and freeze-dried.

### 2.4. Surface Topography and Properties

The surface topography and size of the composite nanofibrous scaffolds was examined by a Hitachi-4800 scanning electron microscope (SEM) (Hitachi India Pvt. Ltd, Mumbai, India). Before examination, the samples were adhered to SEM studs, and coated with 5 nm thin gold film using a sputter-coater (EMITECH K550X, EMITECH ENGINEERING (INDIA) PRIVATE LIMITED, Mumbai, India) for 4 min at 30 mA. The fibers were quantified using DiameterJ tool in imageJ (https://imagej.net/DiameterJ) and plot was generated into origin software.

### 2.5. Quantitative Analysis of rhEGF-Immobilized Composite Nanoscaffolds

The amine groups present on the surface of 1,6-hexanediamine treated nanoscaffolds (diameter 15 mm) were determined using ninhydrin assay [39,40]. Ninhydrin solution (1.0 M) was prepared in ethanol and each scaffold was immersed into this solution for 1 min. Then, the scaffolds were transferred to a glass vial that was heated for 15 min at 70 °C. The scaffolds were then dissolved in 2 mL tetrahydrofuron and isopropanol each and mixed with the solution. The absorbance was measured at 560 nm using a Lambda 25 UV−Vis spectrophotometer (Perkin-Elmer, New York, NY, USA). The amine groups on the scaffolds were quantified by plotting a standard curve of 1,6-hexanediamine solutions in isopropanol in the range of 5 to 25 μg/mL. To determine the amount of immobilized rhEGF, the fluorescence intensity of the tryptophan group of rhEGF immobilized on the nanofibrous scaffolds was measured. The rhEGF assay was performed using a fluorescence spectrophotometer (Perkin Elmer Model–LS 45, Perkin Elmer, New York, NY, USA) at 280 nm excitation and 342 nm emission wavelength as the intrinsic tryptophan fluorescence. The amount of immobilized rhEGF was determined by subtracting rhEGF residual intensity in solution at the end of the immobilization process from the initial amount of rhEGF in the solution.

### 2.6. Thermal Analysis

The thermal properties of composite nanoscaffolds were determined using thermogravimetric analysis (TGA) and differential scanning calorimetry (DSC). TGA was performed in an automatic thermal analyzer system Thermogravimetry/Differential Thermal Analysis (TG/DTA) (Diamond TG/DTA 8.0, Perkin-Elmer, Perkin Elmer India Ltd., Noida, India). Sealed samples were healed in an aluminum pan at 20.00 °C/min from 50.00 °C to 900.00 °C under a constant nitrogen flow rate of 150 mL/min through the sample chamber. The DSC measurements were carried out in an automatic thermal analyzer system (Diamond TG/DTA 8.0, Perkin-Elmer, New York, NY, USA) in the temperature range from −50 °C to 300 °C at a heating rate of 15 °C /min under a nitrogen atmosphere.

### 2.7. X-Ray Diffraction

The crystalline structure of composite nanoscaffolds was characterized by Spinner PW3064 XPERT-PRO diffraction (XRD) system using Cu Kα radiation with continuous scanning at 40 mA, 45 kV. The scan was performed from 5° to 60° (2θ). The plane spacing of different diffraction planes (d_hkl_) can be calculated from the Bragg’s Law (Equation (1)):

d_hk_ = λ/2 sinθ ^ (1)

where λ is the wavelength of the copper anode source (λ = 1.54 Å), and θ stands for the diffraction angle of each indexed diffraction plane.

### 2.8. Degree of Swelling and Porosity.

The degree of swelling of nanoscaffolds was calculated by immersing them in phosphate buffer saline (pH 7.4) for 24 h at room temperature [21]. After 24 h, the nanoscaffolds were removed, and the excess buffer solution was wiped with filter paper and weighed. The weights were recorded, and the swelling index was calculated using Equation (2) as given below.

(2) $$\mathrm{Degree}\;\mathrm{of}\;\mathrm{swelling}\;(\%)=\frac{W-W_{O}}{W_{O}}\times 100$$

where, W = weight of scaffolds after immersion and Wo = weight of scaffolds before immersion: The porosity of the composite nanoscaffolds was measured using the gravimetric method [21]. The thicknesses of the nanoscaffolds were measured using microcallipers, and then their apparent density (ρ_scaffold_) and porosity(ε) were calculated according to the Equation (3).

(3) $$\varepsilon =1-\frac{\rho_{\mathrm{scaffold}}}{\rho_{\mathrm{material}}}$$

where, ρ_scaffold_ is the density of scaffold and ρ_material_ is the density of bulk PLGA

### 2.9. Water Contact Angle Measurement

The water contact angle measurements were performed using an FTA1000 (First Ten Ångstroms Inc., Manchester, UK) instrument. An average obtained from at least five measurements of the contact angle was obtained with MilliQ water (volume ~3 nano-liters, FirstTenÅngstroms Inc., Noida, India) on different nanoscaffolds composite spots. For statistical validation of results, each measurement of a particular contact angle was recorded in 150 images taken within 5 s with a Pelco Model PCHM 575-4 camera (standard deviation ~2°, unless otherwise stated). Images analysis was performed by the FTA Windows Mode 4 software.

### 2.10. In Vitro Biodegradability Studies

To evaluate the in vitro biodegradability of composite nanoscaffolds, degradation studies were performed over a period of 14 days in phosphate buffer saline solution (PBS; pH 7.4) [21,41]. Degradation, as the percentage of weight loss (*W_L_*) was calculated on 3rd, 7th, 10th, and 14th day using Equation (4).

(4) $$W_{L}\left(\%\right)=\frac{W-W_{O}}{W_{O}}\times 100$$

where *W_O_* is initial weight and *W* is weight after degradation:

### 2.11. Drug-Polymer Profile

Drug assay was carried out to determine the drug entrapment efficiency of the composite nanoscaffolds as reported in our previous study [21]. Drug loaded nanoscaffolds were placed in phosphate buffer saline (pH 7.4) and centrifuged at 8000 rpm for 5 min. A 3 mL of ninhydrin reagent was added to the supernatant, and the absorbance was measured using a Lambda 25 UV−vis spectrophotometer (Perkin-Elmer, New York, NY, USA) at 566 nm. The amount of drug in the sample was calculated using the standard curve prepared from PBS. The drug entrapment efficiency was calculated by comparing the amount of drug used to prepare the scaffolds according to Equation (5).

(5) $$\mathrm{Drug}\;\mathrm{entrapment}\;\mathrm{efficiency}\;\left(\%\right)=\frac{M}{M_{O}}\times 100$$

where, M = mass of entrapped drug and M_O_ = mass of drug used in the formulation. The drug loading capacity is the ratio of the mass of bound drug to the mass of the scaffold and was calculated using Equation (6).

(6) $$\mathrm{Drug}\;\mathrm{loading}\;\mathrm{capacity}\;\left(\%\right)=\frac{F}{W}\times 100$$

### 2.12. In Vitro Drug Release Studies

The release of gentamicin sulfate from composite nanoscaffolds was studied at different pH values. It is well known that the pH values have a significant impact on the assembly and organization of nanoscaffolds by affecting the surface charge density of weak electrolytes. It also has an effect on the degree of ionization of weak polyelectrolytes [42]. The membrane diffusion technique was used for the in-vitro release studies of gentamicin sulfate from composite nanoscaffolds, as described in our previous study [21,41]. The studies were conducted within a temperature controlled incubator (37 ± 0.5 °C) using PBS (150 mM, pH 7.4) under gentle mixing.

The nanoscaffolds were incubated in 5 mL of aqueous buffer solution with pH 3, 7.4, and 9, respectively. Aliquots of 1.0 mL of the diffusion medium were withdrawn at certain incubation time points and were replaced with an equal quantity of a fresh diffusion medium. Samples were quantitatively analyzed using Systronics 10 UV–Vis spectrophotometer at 566.0 nm, after treatment with ninhydrin reagent against the mixture of diffusion medium and ninhydrin reagent as blank.

### 2.13. In Vitro Antibacterial Activity Testing

Antibacterial activity of composite nanoscaffolds was evaluated against *Staphylococcus aureus*, a Gram-positive bacteria in liquid medium [21,24]. *Staphylococcus aureus* was obtained from the Microbiology department, Sam Higginbottom Institute of Agriculture, Technology and Sciences, Allahabad. The bacterial culture was grown in nutrient broth, and when the absorbance reached 0.1 to 0.2 at 625 nm, 5 mL of the culture was taken in test tubes, and the drug loaded nanoscaffolds were submerged in them. GS powder was taken as a positive control while the tube without scaffold was taken as negative control. Another tube with cotton gauze was set as another negative control. The samples were incubated on a shaker at 200 rpm for 24 h at 37 °C. After that, the absorbance at 625 nm was monitored using UV−vis spectroscopy. All experiments were performed in triplicate, and only the average values were reported. The percentage of bacterial inhibition was calculated from Equation (7).

(7) $$\mathrm{Bacterial}\;\mathrm{inhibition}\left(\%\right)=\frac{I_{C}-I_{S}}{I_{C}}\times 100$$

where Ic and Is are the average absorbance readings of the control group and the experimental group, respectively.

The assessment was conducted based on the disc agar diffusion method [21,24]. Nutrient agar plates were prepared by autoclaving the nutrient agar media and pouring onto petri dishes and air-dried. A 100 µL aliquot of bacteria reconstituted in nutrient broth and previously subcultured was spread onto an agar plate. The GS-free and GS-containing nanoscaffolds were cut into circular discs (15 mm in diameter) and placed on the top of the agar plate. The plates were incubated at 37 °C for 24 h. If inhibitory concentrations were reached, there would be no growth of the bacteria, which could be seen as a clear zone around the scaffold specimens. The zone was then recorded as an indication of inhibition against the bacterial species.

### 2.14. In Vivo Wound Healing Studies in Diabetic Mice

In vivo studies were performed in accordance with the Institutional Animal Ethics Committee (IAEC) constituted as per directions of the Committee for the Purpose of Control and Supervision of Experiments on Animals (CPCSEA), under the Ministry of Animal Welfare Division, Government of India, New Delhi. The approval from the Institutional Animal Ethical Committee of Bundelkhand University, Jhansi, was taken before the experimental work (BU/Pharm/IAEC/13/29). Five groups of Female C57BL/6 mice weighing (14–15 g) were selected, each group having 6 mice. All animals were allowed to adapt to cages for 3 days, after which they were fasted overnight. Streptozotocin (STZ) was freshly dissolved in (0.1 M, pH 4.5) citrate buffer and non-insulin-dependent diabetes mellitus was induced in overnight fasted mice by a single intraperitoneal injection of Streptozotocin (40 mg/kg bodyweight). Blood glucose levels were measured 4 days after STZ injection, and only mice with fasting blood glucose levels greater than 200 mg/dL were considered to be diabetic and were used in the experiment. Wounds in the diabetic mice were created by following the excision wound model, whereby their dorsal hairs were shaven, and an excision made with scissors and tweezer on the back of the animal. The wound areas were sterilized with povidone iodide. All treatments started 4 days after STZ injection. For determination of wound healing activity, the following treatments were applied after creating wounds on five groups—Group 1 (C)—no treatment (negative control), Group 2 (G)—pure GS solution (positive control), Group 3 (P)—PLGA/Gelatin 70:30 scaffold without GS and rhEGF, Group 4 (PG)—PLGA/Gelatin 70:30 scaffold loaded with GS and rhEGF both and Group 5 (PG)—PLGA/Gelatin 70:30 scaffold loaded with GS only. The treatments were applied to aseptically treated wounds to determine the therapeutic effects on the wounds. After each treatment interval, the scaffolds were removed with tweezers. The wounds were photographed on day 1 and the treatment regime repeated on day 4, 8, and 12. The percentage reduction of the wound area was calculated using Equation (8) below.

(8) $$\mathrm{Wound}\;\mathrm{area}\;\left(\%\right)=\frac{A}{A_{i}}\times 100$$

where, A_i_ is the initial wound area, and A is the wound area after a fixed time interval.

### 2.15. Statistical Analysis

Data are expressed as means ± standard deviation. For the statistical validation of data, three independent experiments (n = 3) were performed in triplicates. Statistical analysis was conducted using a paired *t*-test or an unpaired, two-tailed Student’s *t*-test. A *p*-value of <0.05 was considered to be statistically significant.

## 3. Results and Discussion

### 3.1. Surface Topography and Properties of Composite Nanoscaffolds

**Figure 2:**
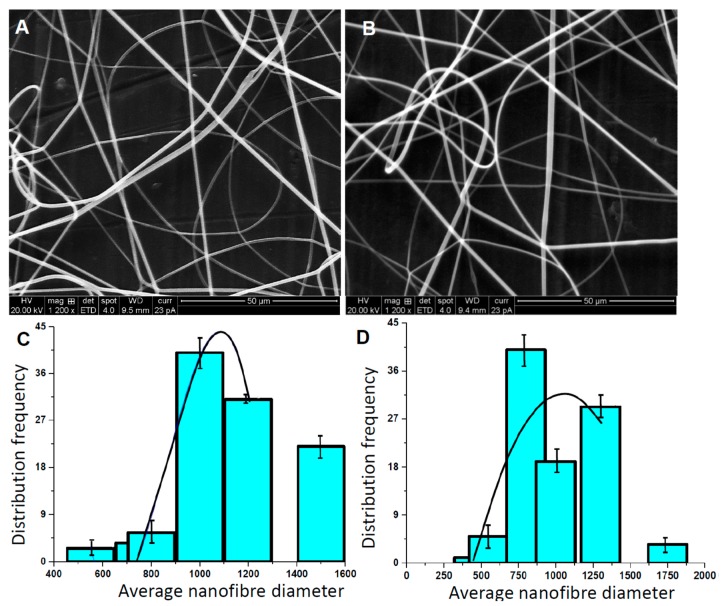
Physical Characterization. Scanning electron microscopy (SEM) micrographs of (**A**) PLGA/Gelatin 70:30 (**B**) PLGA/Gelatin 50:50 nanofibrous scaffolds and (**C**) Average fiber size distribution in PLGA/Gelatin 70:30 and (**D**) PLGA/Gelatin 50:50 nanofibrous scaffolds. The results are expressed as the median ± standard deviation obtained from analysis of 20 to 25 images for each electrospun polymer film (the black curve is drawn to shows the Gaussian distribution of frequency).

The morphologies of the PLGA/Gelatin 70:30 and PLGA/Gelatin 50:50 nanofibrous scaffolds as depicted by SEM micrographs are shown in Figure 2A,B, respectively.

The corresponding average fiber diameters are shown as a bar plot in Figure 2C,D, respectively. The graph indicates that there is a random distribution of fibers in both, PLGA/Gelatin 70:30 and 50:50 nanoscaffolds. The average fiber diameter in PLGA/Gelatin 70:30 is slightly higher than 50:50 (~750 nm to 900 nm). The fibers exhibit ECM like interconnectivity, and no bead formation was observed. As gelatin adds viscosity to the solution, both types of fibers were smooth and without any breakage. The probability of fiber breakage increases with increasing gelatin content since an increase in gelatin content decreases the viscosity of the polymeric solution. The calculated average diameters of PLGA/Gelatin 70:30 and PLGA/Gelatin 50:50 nanofibers were 589.6 nm and 566.0 nm, respectively. As the concentration of gelatin increased, the diameter of the nanofibers decreased. This may be attributed to the fact that on increasing the gelatin content, the viscosity of the composite solution decreased, and the amino acids of the gelatin improved the self-repulsion and stretching force due to the increasing charge density of the jet during the electrospinning process. This resulted in the smaller fiber diameter [43]. The overall structure of the fibers mimics that of the ECM.

### 3.2. Quantitative Analysis of rhEGF-Immobilized Nanoscaffolds

**Figure 3:**
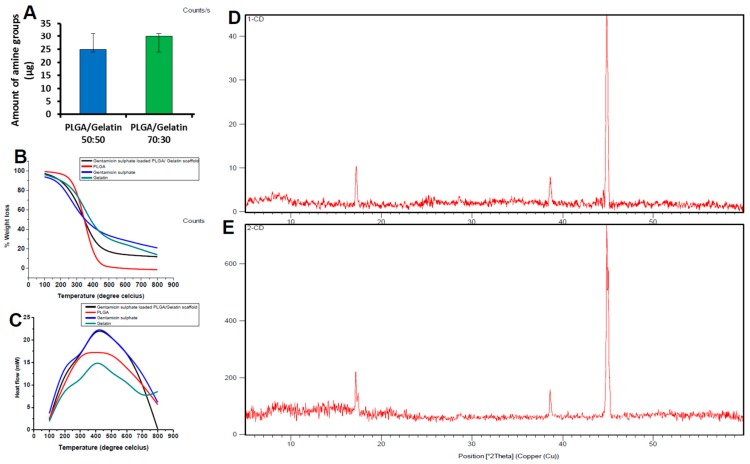
Biochemical characterization. (**A**) 2,2-dihydroxyindane-1,3-dione (ninhydrin) assay applied to 1,6 hexanediamine treated nanofibrous scaffolds (the results are expressed as the median ± standard deviation). (**B**) Thermogravimetric analysis (TGA) curves of PLGA, Gentamicin sulfate loaded PLGA 70:30 nanofibrous scaffolds, Gentamicin sulfate and Gelatin. (**C**) Differential scanning calorimetry (DSC) curves of PLGA, Gentamicin sulfate loaded PLGA nanofibrous scaffolds, Gentamicin sulfate and Gelatin. (**D**) X-Ray diffraction (XRD) spectra of (**A**) PLGA/gelatin 70:30 and (**E**) PLGA/gelatin 50:50 nanofibrous scaffolds.

The number of amine groups (μg per scaffold) on scaffolds treated with ninhydrin was quantified using the standard curve, and the results are shown in Figure 3A. It can be inferred from the graph that the quantified amount of amine groups were 25 µg and 30 µg per scaffold in PLGA/Gelatin 70:30 and PLGA/Gelatin 50:50 nanoscaffolds, respectively. The presence of amine groups in both the scaffolds clearly indicates the success of aminolysis. The results obtained from fluorescence spectrophotometry confirmed the presence of rhEGF on the surface of the nanofibrous scaffolds. The rhEGF-conjugated PLGA/Gelatin 50:50 and PLGA/Gelatin 70:30 nanoscaffolds had 7.46 µg and 2.92 µg of rhEGF, respectively, per scaffold when 10 µg of rhEGF was employed for a chemical reaction. Thus, the rhEGF immobilization efficiencies reached 74.6% and 29.2% for PLGA/Gelatin 50:50 and PLGA/Gelatin 70:30 nanofibrous scaffolds, respectively. It could be attributed to the fact that since the number of amine groups per scaffold was higher in PLGA/Gelatin 50:50 scaffolds, they were able to immobilize a greater amount of rhEGF than the PLGA/Gelatin 70:30 nanofibrous scaffolds.

### 3.3. Thermal Analysis

The thermal properties of composite nanoscaffolds and other supplements were characterized using TGA and DSC. TGA curves are shown in Figure 3B. The results from the TGA showed two significant weight losses for the gentamicin sulfate at 220 °C and 330 °C. The TGA curves of raw PLGA showed weight losses at 210 °C and 400 °C. In contrast, the TGA curve of gentamicin loaded PLGA/gelatin 70:30 nanofibrous scaffolds showed single significant weight loss at about 260 °C, respectively. As the weight loss of gentamicin sulfate and pure PLGA started at lower temperature compared to the gentamicin sulfate loaded scaffolds, it showed better thermal stability of the composite scaffolds in comparison to gentamicin sulfate or pure polymer alone. Due to the formation of covalent bonds between gentamicin and PLGA/gelatin scaffolds, the labile oxygen containing functional group availability was decreased. Less than 2% weight loss occurred below 200 °C. In addition, the slow weight loss of about 40% below 260 to 350 °C that may be due to the loss of residual functional group on the gentamicin-loaded scaffolds. These observations were missing in the negative control without the gentamicin.

The DSC thermograms of PLGA/Gelatin 70:30 nanofibrous scaffolds are shown in Figure 3C. A peak was observed at the temperature of 210 °C on the DSC thermogram of pure gentamicin sulfate powder, corresponding to the melting point of 210 °C. Another peak was seen at 410 °C, which was due to the reaction between the gentamicin sulfate structures, i.e., degradation of gentamicin sulfate. In the pure PLGA polymer, no sharp peak was observed which may be attributed to the amorphous nature of the PLGA. Whereas, the DSC curve of gelatin showed a peak at 405 °C. In contrast, in the DSC curves of gentamicin loaded PLGA/gelatin 70:30 nanofibrous scaffolds, the peak occurred at 430 °C. This clearly demonstrates that the stability of the drug is enhanced due to encapsulation of the drug between polymeric chains.

### 3.4. X-Ray Diffraction Analysis

The nanofibrous scaffolds are able to form a stable colloidal layered structure in aqueous solution, that facilitates drug encapsulation. The encapsulation of drug within the PLGA/Gelatin interlayer space may result in a change in the interlayer distance, that can be determined by XRD technology. The XRD patterns of the PLGA/gelatin 70:30 and PLGA/gelatin 50:50 scaffolds after gentamicin sulfate encapsulation are shown in Figure 3D,E, respectively. All the three types of nanofibrous scaffolds displayed patterns lacking any distinct peaks indicating that they are fully amorphous materials. In amorphous materials, X-rays will be scattered in many directions leading to a large bump distributed in a wide range (2 Theta) instead of high intensity narrower peaks. Appearance of the broad peaks in the 2θ = 5–40° region was attributed to the amorphous nature of the PLGA. The absence of any distinct peaks of crystalline gentamicin sulfate in the XRD spectra indicates that the drug is no longer present as crystalline material, but had been totally converted to an amorphous state. The XRD data suggested that the incorporation of gentamicin sulfate within polymers is primarily via the drug intercalation within the polymer interlayer space.

It is also possible that a small portion of gentamicin sulfate can be adsorbed onto the polymer surface via hydrogen bonding or other weak forces. The XRD spectrum of the scaffolds displayed two peaks at 19.3° and 23.5° which were located at the same position with the PEG 400. The sharp XRD peaks indicate the crystallization of PEG in the scaffolds.

### 3.5. Degree of Swelling and Porosity Analysis

**Figure 4:**
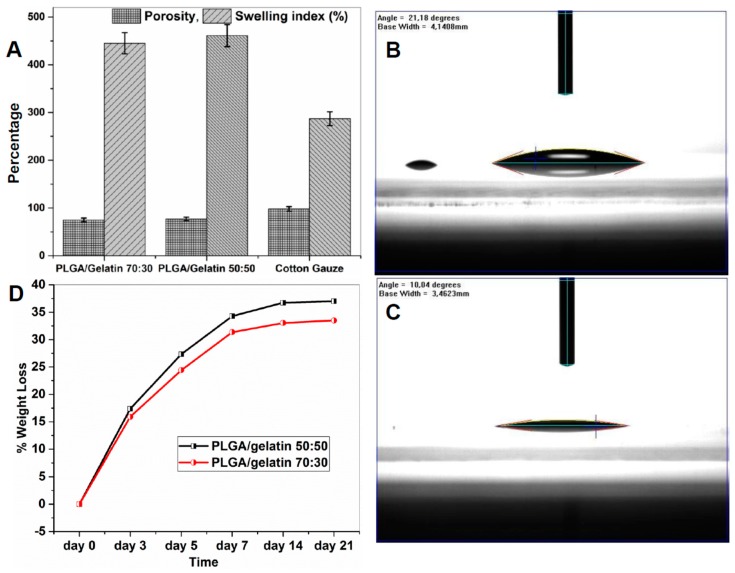
Scaffold stability and wettability analysis. (**A**) Comparison of swelling index and porosities of electrospun PLGA/gelatin 70:30 and 50:50 nanofibrous scaffolds (the results are expressed as the median ± standard deviation from five samples). (**B**) Water Contact Angle Value for PLGA/Gelatin 50:50 Nanofibrous Scaffold. (**C**)Water Contact Angle Value for PLGA/Gelatin 70:30 Nanofibrous Scaffold. (**D**) Biodegradability characteristics of PLGA/gelatin 50:50 and PLGA/gelatin 70:30 nanofibrous scaffolds.

The gentamicin sulfate loaded PLGA/Gelatin 70:30 and PLGA/ Gelatin 50:50 nanofibrous scaffolds were analyzed to determine the swelling behavior by incubating them in PBS (pH 7.4) for 24 h at 37 ºC. The results are represented graphically in Figure 4A. The swelling ratios of the PLGA/Gelatin 70:30 and PLGA/ Gelatin 50:50 nanofibrous scaffolds were 421.33 ± 8.22% and 445.33 ± 4.99%, respectively. The swelling index of cotton gauze was only 280.00 ± 8.64%. It can be inferred from these results that the higher swelling index of PLGA/ Gelatin 50:50 scaffolds was due to more concentration of gelatin as compared to the PLGA/Gelatin 70:30 scaffolds, as gelatin improves the hydrophilicity of the scaffold. This may be due to the amine and carboxylic functional groups present in the gelatin structure [44]. The obtained swelling index enables the prepared scaffolds to be used as wound dressings for absorbing excess exudates even from deep wounds with high amounts of exudates [44,45].

Porosity is a very important criteria for the application of nanofibrous scaffolds in tissue engineering and wound healing practices because the microscale and nanoscale porous structure are most suitable for convenient passage and exchange of nutrients and gases, that are important for cellular growth and tissue regeneration [46]. Porosity should be in the range of 60 to 90% for cellular penetration and efficient tissue regeneration [47]. In the present work, porosity was calculated by the gravimetric method and the results are represented graphically in Figure 4A. The porosities of PLGA/Gelatin 50:50 and PLGA/Gelatin 70:30 nanofibrous scaffolds were 74.1% and 77%, respectively. In contrast, the porosity of cotton gauze was 102%. The possible reason could be that the increased gelatin content decreased the fiber diameter, which caused the decreased porosity of scaffolds.

### 3.6. Water Contact Angle Measurements

Generally, if the water contact angle is smaller than 90°, the solid surface is considered hydrophilic and wettable, and if the water contact angle is larger than 90°, the solid surface is considered hydrophobic and not wettable. Figure 4B,C, respectively show that the water contact angle values for PLGA/Gelatin 70:30 and PLGA/Gelatin 50:50 nanofibrous scaffolds were 10.04° and 21.18°, respectively. Hence, it could be concluded that the water contact angles of both the scaffolds was much lesser than 90°. Therefore, the surface of the scaffolds could be considered highly hydrophilic. Moreover, the water contact angle of PLGA/Gelatin 50:50 nanofibrous scaffolds were lower than that of PLGA/Gelatin 70:30 nanofibrous scaffolds due to more amount of Gelatin on the former as compared to the latter.

### 3.7. In Vitro Biodegradability Studies

The in vitro biodegradation studies of the PLGA/Gelatin 70:30 and PLGA/Gelatin 50:50 nanofibrous scaffolds were performed in Phosphate Buffer Saline (PBS; pH 7.4). The scaffolds were placed in a 24-well plate containing 1 mL of PBS in each well and were incubated in vitro at 37 °C for different periods of time (3, 5, 7, 14 days). Figure 4D shows the percent weight losses. A rapid 27.33 ± 1.15% and 24.4 ± 1.82% mass loss was observed in electrospun PLGA/gelatin 50:50 and PLGA/gelatin 70:30 nanofibrous scaffolds, respectively, in first 5 days. After 7 days, the degradability of both scaffolds was decreased which was due to more PLGA in the remaining composition of scaffolds. Since gelatin easily dissolves in water at a temperature of 40 °C, and, hence, by increasing the content of gelatin, the biodegradability of the PLGA/gelatin 50:50 scaffolds was greater as compared to PLGA/gelatin 70:30 scaffolds. In contrast, PLGA nanofibrous scaffolds and cotton gauze used as control did not exhibit any weight loss during the 14 days of incubation.

### 3.8. Drug Loading Capacity and Drug Entrapment Efficiency

Drug loading capacity is the ratio of the mass of bound drug to the mass of the scaffold, whereas, the drug entrapment efficiency is the ratio of the mass of drug released to the mass of total drug added. The mass of gentamicin sulfate loaded per 20 mg of the scaffolds was investigated by UV-Vis spectrophotometry. The results are summarized in Table 1. It was found to be 174.24 µg and 161.68 µg for PLGA/Gelatin 70:30 and PLGA/Gelatin 50:50 nanofibrous scaffolds, respectively. On the other hand, the entrapment efficiency was found to be 87.12% and 80.26% for PLGA/Gelatin 70:30 and PLGA/Gelatin 50:50 nanofibrous scaffolds, respectively. The comparatively higher entrapment efficiency may be due to the fact that PLGA, being a high molecular weight polymer, and the molecular weight further supplemented by gelatin, is able to absorb a comparatively greater amount of active substances. As shown herein, more than 80% entrapment efficiency ensures more than 150 µg of gentamicin sulfate delivered to the wound site for in-situ release. These results are in line with therapeutically used dosages of gentamicin sulfate 100 µg or more, immobilized on the synthetic carrier for successful treatment of infection in mouse model [48].

### 3.9. In vitro Drug Release Studies

**Figure 5:**
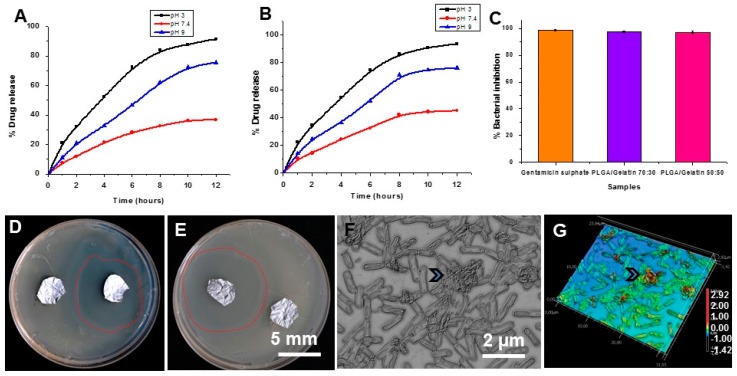
In vitro antibacterial assay. (**A**,**B**) Release profile of gentamicin sulfate on PLGA/Gelatin 70:30 (A) and 50:50 (B) nanofibrous scaffolds at three different pH values. (**C**) Growth inhibition of *Staphylococcus aureus* after treatment with PLGA/Gelatin 70:30, PLGA/Gelatin 50:50 nanofibrous scaffolds and Gentamicin sulfate powder in aqueous media. The results are expressed as the median ± standard deviation from five independent experiments performed in triplicate (n = 5). (**D**) Antibacterial activity of gentamicin sulfate loaded PLGA/ Gelatin 70:30 nanofibrous scaffolds against *Staphylococcus aureus* on LB Agar semisolid media. (**E**) Antibacterial activity of gentamicin sulfate loaded PLGA/Gelatin 50:50 nanofibrous scaffolds against *Staphylococcus aureus* (dotted red line show zone of inhibitions-ZOI around test sample, next to control). (**F**,**G**) Optical and laser scanning micrograph from bacterial cells seeded on Gentamicin Sulfate (GS)/PLGA/Gelatin (50:50) surfaces show sunken morphology of bacterial cells trapped in a network of nanofibers scaffolds (arrow heads).

The gentamicin sulfate release curves from PLGA/Gelatin 70:30 and PLGA/Gelatin 50:50 nanofibrous scaffolds were studied at different pH values, and the release curves are illustrated in Figure 5A,B. The studies from each type of scaffolds revealed constant drug release and no burst effect was observed. It indicates that the drug was homogeneously dispersed in the polymeric matrix and there was no significant amount of drug adsorbed onto the surface of nanofibers. Figure 5A shows the release of gentamicin sulfate from PLGA/Gelatin 70:30 nanofibrous scaffold. At pH 7.4, gentamicin sulfate was released slowly from the PLGA/Gelatin 70:30 nanofibrous scaffold and only 36.64 ± 0.51% of the total bound gentamicin sulfate was released in 12 h. However, 91.46 ± 0.61% and 75.43 ± 0.82% of the drug was released in acidic and basic conditions, respectively, after 12 h. Figure 5B illustrates the release of gentamicin sulfate from PLGA/Gelatin 50:50 nanofibrous scaffold. At pH 7.4, gentamicin sulfate was released slowly from the PLGA/gelatin 50:50 nanofibrous scaffold and only 44.97 ± 0.14% of the total bound gentamicin sulfate was released in 12 h. However, 93.33 ± 0.82% and 76.03 ± 0.75% of the drug was released in acidic and basic conditions, respectively, after 12 h. The release at pH 3 and pH 9 is much higher than that released at pH 7.4. This is so because the hydrogen bonding interaction between gentamicin sulfate and the nanofibrous scaffolds is strongest at the neutral pH. Whereas, at pH 3 and 9, there is a comparatively weaker hydrogen bonding interaction, so the higher amount of gentamicin sulfate was released at pH 3 and 9. There was a higher percentage release of gentamicin sulfate at pH 3 compared to pH 9 because the hydrogen bonding interaction formed under basic conditions was stronger than that under acidic conditions.

### 3.10. In Vitro Antibacterial Activity

The in vitro antibacterial activity of the gentamicin sulfate loaded PLGA/Gelatin 70:30 and PLGA/Gelatin 50:50 nanofibrous scaffolds were explored against *Staphylococcus aureus* M 0092 in aqueous medium. *S. aureus* bacteria are commonly found in diabetic wounds and used as a pathogenic bacterial model for in vitro antibacterial efficacy [49]. Figure 5C illustrates the results of the antibacterial activity assays. The negative control, which was the tube with cotton gauze, completely failed to inhibit bacterial growth. Gentamicin sulfate powder, which was used as the positive control exhibited 98.73 ± 0.68% inhibition of the bacterial growth at the concentration of 5 mg/mL. The PLGA/Gelatin 70:30 and PLGA/Gelatin 50:50 nanofibrous scaffolds showed 97.39 ± 0.62% and 97.20 ± 0.99% inhibition of bacterial growth, respectively. This may be attributed to the active diffusion of the drug molecules from the scaffolds into the liquid medium, thus, inhibiting the growth of bacteria. These results are promising, since the antibacterial activity of the PLGA/Gelatin 70:30 and PLGA/Gelatin 50:50 nanofibrous scaffolds are comparable to that of the pure gentamicin powder, which presents them as a perfect drug releasing vehicle. Thus, the developed nanofibrous scaffolds could be used as a potential wound dressing material that would successfully eliminate infections, thereby, accelerating wound healing.

The antibacterial activity of the PLGA/Gelatin 70:30 and PLGA/Gelatin 50:50 nanofibrous scaffolds was also analyzed over a solid medium. Figure 5C,D shows the digital photographs of the zone of inhibition on nutrient agar plates. The gentamicin sulfate loaded PLGA/Gelatin 50:50 and PLGA/Gelatin 70:30 nanofibrous scaffolds had “zone of inhibition” values of 40 mm (Figure 5B) and 41 mm (Figure 5C), respectively. These results suggested that each nanofibrous scaffold had a good bacterial inhibition efficacy under the studied conditions. In contrast, PLGA/Gelatin nanofibrous scaffolds without gentamicin sulfate encapsulation did not inhibit the bacterial growth, suggesting that the bacterial inhibition effect is solely related to the encapsulated Gentamicin sulfate drug. Hence, the gentamicin sulfate loaded nanofibrous scaffolds were bactericidal to the testing microorganism due to the strong antibacterial ability of gentamicin sulfate. The results indicate that gentamicin sulfate loaded scaffolds possess efficient antibacterial property and can be effectively used in the treatment of wound healing or dermal bacterial infections, thereby, proving a potential application for use as a drug delivery and as a wound dressing agent [50].

We also studied the morphologies and biovolume of bacterial cells seeded on two nanofibrous scaffold supplemented with GS using laser scanning microscope to quantify surface and biovolume (Figure 5F–G and Supplementary Figure S2A–E). As seen in optical and 3D height profile images, the bacterial cells are entangled into fibers, which affect bacterial cells surface into sunken morphology, reducing their biovolume compare with untreated control cells showing flagella and normal morphology (arrow, Figure 5F–G and Figure S1C).

### 3.11. In Vivo Wound Healing Activity on Diabetic Mice and Organ Toxicity Evaluation

**Figure 6:**
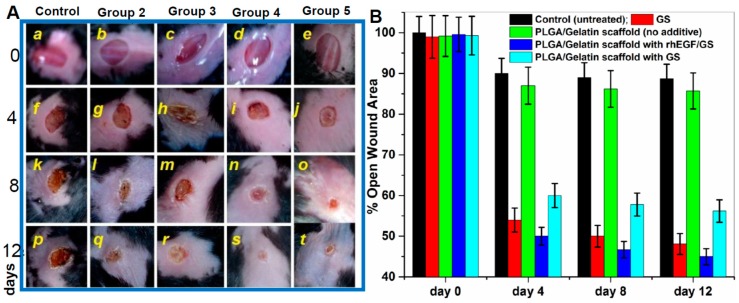
In vivo wound healing assay in mouse model. (**A**) Wound Healing in Diabetic C57/BL6 Mice after Treatment with Various Electrospun Nanofibrous Scaffolds (Batch 1) [a–t represent the extent of wound healing during 12 days period] Group 1—No treatment (negative control); Group 2—0.1% gentamicin sulfate ointment (positive control); Group 3—PLGA/Gelatin 70:30 scaffold without gentamicin sulfate and rhEGF; Group 4—PLGA/Gelatin 70:30 scaffold with gentamicin sulfate and rhEGF; Group 5—PLGA/Gelatin 70:30 scaffold with gentamicin sulfate and without rhEGF. (**B**) Open wound area (% of initial area) over 12 days for each treatment group from control and three experimental groups performed from five different wound healing assays. The results are expressed as the median ± standard deviation from five independent experiments performed in triplicate (n = 5).

Animals with induced diabetes were confirmed with diabetic symptoms and were subjected to wound healing treatments with the gentamicin-rhEGF-nanofibrous scaffolds and controls. The wound area was measured daily from day 0 to 12 and wounds were monitored qualitatively (Figure 6A), and quantitatively (Figure 6B) for the wound closure to assess the efficacy of therapeutics designed. The results revealed that rhEGF immobilized nanofibrous scaffolds significantly increased the wound closure rates compared to other treatments. The representative images at 1, 4, 8, and 12 days after treatment with rhEGF-PLGA/Gelatin 70:30 nanofibrous scaffold, PLGA/gelatin 70:30 nanofibrous scaffold with gentamicin sulfate, PLGA/gelatin 70:30 nanofibrous scaffold without rhEGF, untreated wound and gentamicin sulfate are shown in Figure 6A. Specifically, the animals with PLGA/Gelatin 70:30 nanofibrous scaffolds with rhEGF and gentamicin sulfate showed 19.13 ± 5.68% open wound area on the 4th day (Figure 6A), 12.99 ± 1.17% open wound area on the 8th day (Figure 6An), and 3.25 ± 2.95% open wound area on the 12th day (Figure 6As), which were much less than other treatments. The data represented the mean ± S.D. for six mice per group.

**Figure 7:**
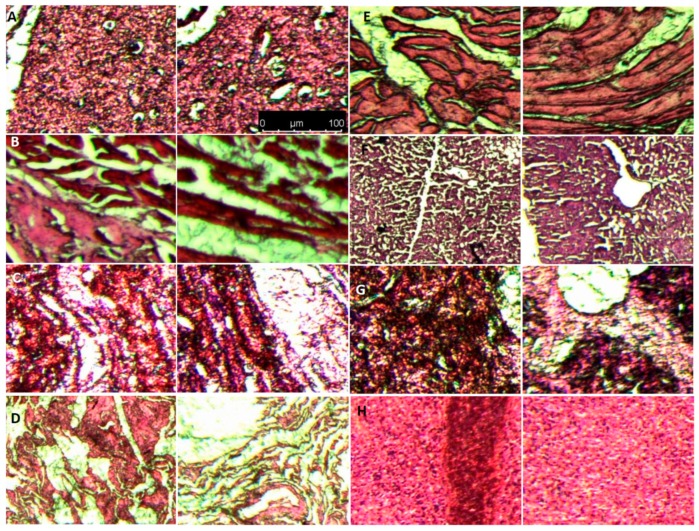
Histological evaluation of In vivo *organ* toxicity with histopathological staining on organ sections. (**A**) Brain (**B**) Heart (**C**) Kidney (**D**) Lung (**E**) Muscle (**F**) Liver (**G**) Pancreases (**H**) Spleen. Each image from **A**–**H** has control (left panel) and scaffold applied (right panel) sample section with Hematoxylin and Eosin (**H**,**E**) staining.

We also took samples from different organs of mice treated with GS/PLGA/gelatin 50:50 after day 12 and compared with the controls group without any treatment. As shown in Figure 7, histopathological examination results showed that there was no apparent change in different organs morphology and no other abnormal changes in the mice tissue in control versus experimental group. This confirms that the nanofibrous scaffolds, thus, prepared are biocompatible and do not cause any side effects. These results open new opportunities for rapid wound healing in different vascular lesions as advance therapeutics, which is sought as a major challenge in current nanomedicine in vascular biology [51,52].

## 4. Conclusions

In summary, a novel nanocomposite carrier device composed of PLGA/Gelatin 70:30 and PLGA/Gelatin 50:50 nanofibrous scaffolds loaded with gentamicin sulfate and immobilized with rhEGF was developed for diabetic wound healing applications. The developed nanofibrous scaffolds resembled the morphology and architecture of the native extracellular matrix (ECM), which makes them useful as a suitable wound-healing scaffold. The gentamicin sulfate stability and bactericidal properties were not affected by the encapsulation process and release. The rhEGF accelerated wound-healing rates at the initial stage of the healing process. The results obtained are promising, since the simultaneous delivery of antibiotic and bioactive molecule from a nanocarrier material to inactivate bacteria in diabetic wounds and hasten the wound healing at the same time was clearly demonstrated. The nanocomposite has the potential to synergistically improve the impaired wound healing in diabetic patients via combined antibacterial activity and rhEGF supply.

## Figures and Tables

**Table 1:** Drug loading capacity and drug entrapment efficiency of nanofibrous scaffolds.

Nanofibrous Scaffold	Drug Loading Capacity (µg)	Drug Entrapment Efficiency (%)
PLGA/Gelatin 50:50	161.68 ± 13.2	80.26 ± 6.9
PLGA/Gelatin 70:30	174.24 ± 19	87.12 ± 8.1

## Supplementary Materials

The following are available online at https://www.mdpi.com/1999-4923/11/4/180/s1, Figure S1: The SEM of nanofibrous scaffolds after gentle rinsing and dehydration. Figure S2: Quantitative analysis of bacterial biovolume on nanofibrous scaffolds. (A-B) 3D height map and optical micrograph of control bacterial cells showing normal morphology and flagella (C). (D-E) Example of extracting individual bacterial biovolume from the optical images (cells shown in dotted red line in D as an example) to quantify the biovolume of bacteria on different surfaces.
