# Supplementary material for: In Vivo Biocompatibility of Electrospun Biodegradable Dual Carrier (Antibiotic + Growth Factor) in a Mouse Model—Implications for Rapid Wound Healing
Source: Pharmaceutics. 2019 Apr 14;11(4):180. doi: 10.3390/pharmaceutics11040180 (PMC6523103; doi:10.3390/pharmaceutics11040180)
Supplement: Supplementary file 1 [file pharmaceutics-11-00180-s001.pdf]

Supplementary Information

In vivo biocompatibility of an electrospun, biodegradable dual-carrier containing antibiotic and growth factor in a mouse model - Implications for rapid wound healing

[1,2]Charu Dwivedi, Himanshu Pandey[2,3], Avinash C. Pandey[2], Sandip Patil[4], Pramod W. Ramteke*[1] Peter Laux[5], Andreas Luch[5] and Ajay Vikram Singh*[5,6]

[1]Department of Biological Sciences, Sam Higginbottom University of Agriculture, Technology and Sciences, Allahabad- 211007 (India).

[2]Nanotechnology Application Centre, Faculty of Science, University of Allahabad, Allahabad- 211002 (India).

[3]Department of Pharmaceutical Sciences, Faculty of Health Sciences, Sam Higginbottom University of Agriculture, Technology & Sciences, Allahabad- 211007 (India)

[4]E-Spin Nanotech Pvt Ltd, Kanpur- 208016 (India).

[5]Department of Chemical and Product Safety, German Federal Institute for Risk Assessment (BfR), Max-Dohrn-Strasse 8-10, 10589, Berlin, Germany

[6]Physical Intelligence Department, Max Planck Institute for Intelligent Systems, 70569 Stuttgart, Germany.

*Corresponding author email: *Ajay Vikram Singh- Ajay-Vikram.Singh@bfr.bund.de, Charu Dwivedi* charucas0505@gmail.com; Pramod W. Ramteke* pwramteke@gmail.com*.

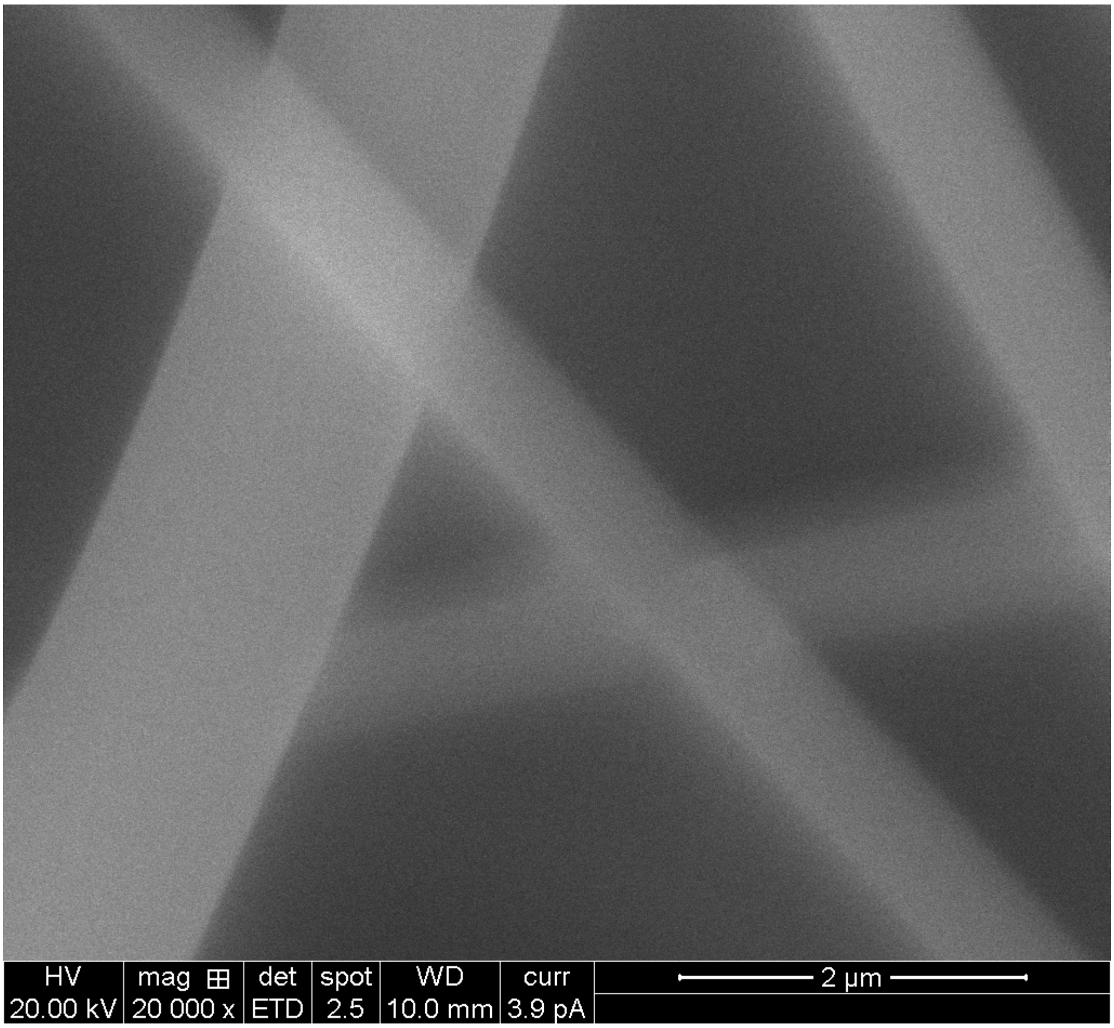

Figure S1. The SEM of nanofibrous scaffolds after gentle rinsing and dehydration.

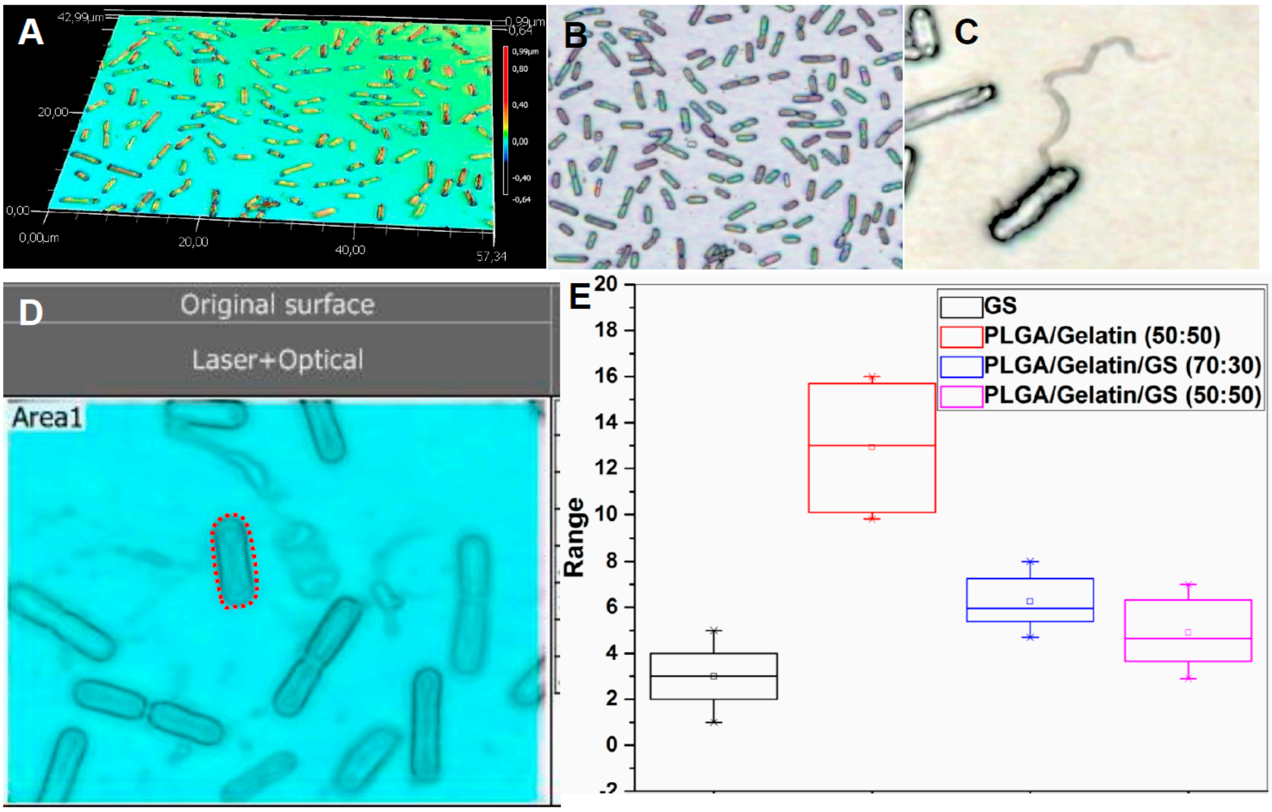

Figure S2. Quantitative analysis of bacterial biovolume on nanofibrous scaffolds. (A-B) 3D height map and optical micrograph of control bacterial cells showing normal morphology and flagella (C). (D-E) Example of extracting individual bacterial biovolume from the optical images (cells shown in dotted red line in D as an example) to quantify the biovolume of bacteria on different surfaces.
